# A case of pterygium-like proliferation containing postoperative limbal dermoid remnants: a clinicopathological study

**DOI:** 10.1186/s12886-020-01767-5

**Published:** 2021-01-06

**Authors:** Mizuho Mitamura, Satoru Kase, Takeshi Ohguchi, Susumu Ishida

**Affiliations:** 1 Department of Ophthalmology, Faculty of Medicine and Graduate School of Medicine, Hokkaido University, N-15, W-7, Kita-ku, Sapporo, 060-8638 Japan; 2 Department of Ophthalmology, Teine Keijinkai Hospital, Sapporo, Japan

**Keywords:** Pterygium, Limbal dermoid, Histopathology, Ki67

## Abstract

**Background:**

This study reports a case of pterygium-like proliferation containing postoperative limbal dermoid remnants and its clinicopathological features.

**Case presentation:**

A 79-year-old Japanese woman, with a history of congenital limbal dermoid resection at age 12, presented with a pterygium-like tissue growth in the left eye. Its temporal location and marked thickness with abundant fatty droplets were atypical of primary pterygium. We performed pterygium surgery and ocular surface reconstruction. Pathological findings included squamous metaplasia, neovessels, and elastic degeneration, as well as prominent subepithelial and stromal accumulation of collagen fibers, adipose tissue formation, and presence of a peripheral nerve corresponded with the frequent findings of limbal dermoid. Ki67, a marker for cell proliferation, was immunopositive in pterygial epithelial cells and neovascular endothelial cells, but not in dermoid components.

**Conclusions:**

Although the pathological finding of degenerative elastic fibers indicated the common feature of ultraviolet-induced pterygium, clinical appearances were atypical possibly due to modification with dermoid remnants.

## Background

Pterygium is a triangular fibrovascular proliferation that usually extends from the nasal conjunctiva and encroaches upon the cornea. Histopathologically, primary pterygium is characterized by epithelial proliferation, epithelial-mesenchymal transition, and an activated fibroblastic stroma with inflammation, neovascularization, and matrix remodeling [1]. We demonstrated that proliferation activity was higher in pterygial epithelial cells than in normal conjunctival epithelial cells [2]. Elastic degeneration, a common histological finding observed in the stromal tissue of primary pterygium [2], is widely known to be correlated with long-lasting ultraviolet exposure. Pterygium is likely to involve concomitant lesions such as conjunctival benign tumors and conjunctival intraepithelial neoplasia [3].

Limbal dermoid is a congenital benign tumor that presents in a dome shape and consists of various tissues of ectodermal and mesodermal origins [4]. Cases of pseudopterygium formation 2 to 16 months after dermoid excision have been reported [4, 5], however, its pathology remains unknown. We herein report clinicopathological findings of a pterygium-like proliferation with residual limbal dermoid that was incompletely resected several decades ago.

## Case report

**Fig. 1:**
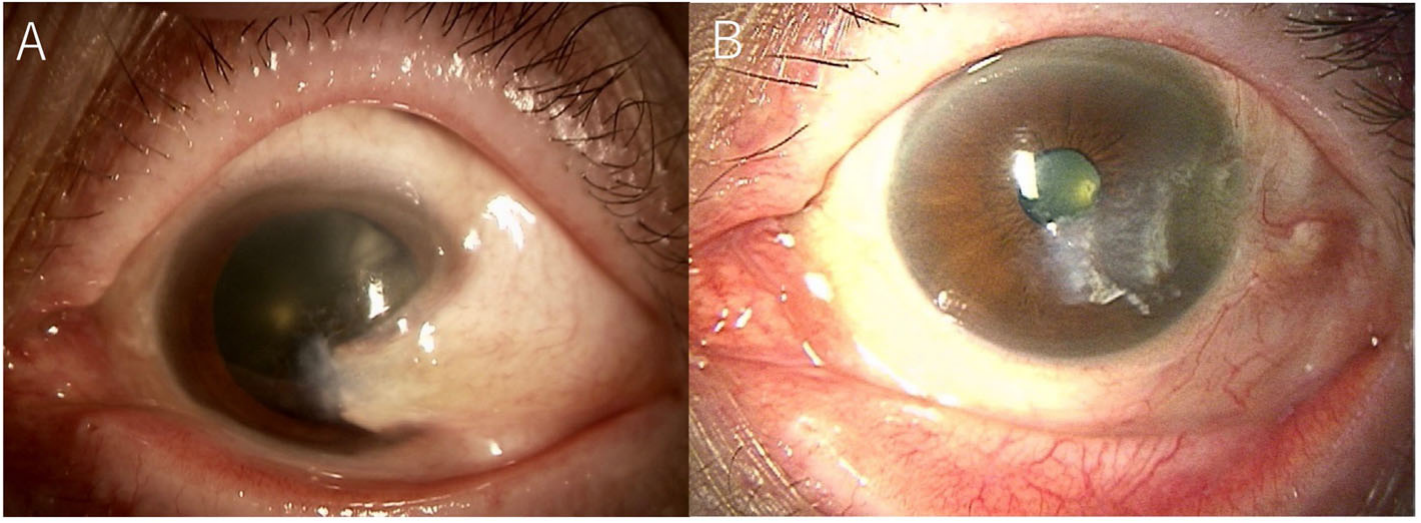
Slit-lamp microscopy of the left eye at the first visit (**a**) and 8 months after pterygium surgery (**b**). **a** The pterygium-like triangular proliferation exhibited with neovessels from the conjunctiva toward the apex of the cornea. Cornea opacity was observed around the head of the tissue. **b** No recurrence of the lesion was observed 8 months after the operation

A 79-year-old Japanese woman complained of blurred vision in her left eye presumably due to a pterygium-like tissue growth. She had a medical history of limbal dermoid from birth, which was removed at 12 years of age. Her decimal best-corrected visual acuity (BCVA) was 1.2 oculus dexter and 0.5 oculus sinister (OS) with hyperopia. Slit-lamp microscopy revealed a markedly thick growth of pterygium-like triangular ocular surface tissue from the temporal conjunctiva toward the apex of the cornea. Corneal opacity was observed around the head of the tissue OS (Fig. 1a). Because of visual impairment with a severe irregular astigmatism, we performed pterygium surgery and ocular surface reconstruction. Eight months after the operation, her BCVA improved to 0.8 OS without obvious recurrence of the lesion (Fig. 1b).

## Histopathological findings

**Fig. 2:**
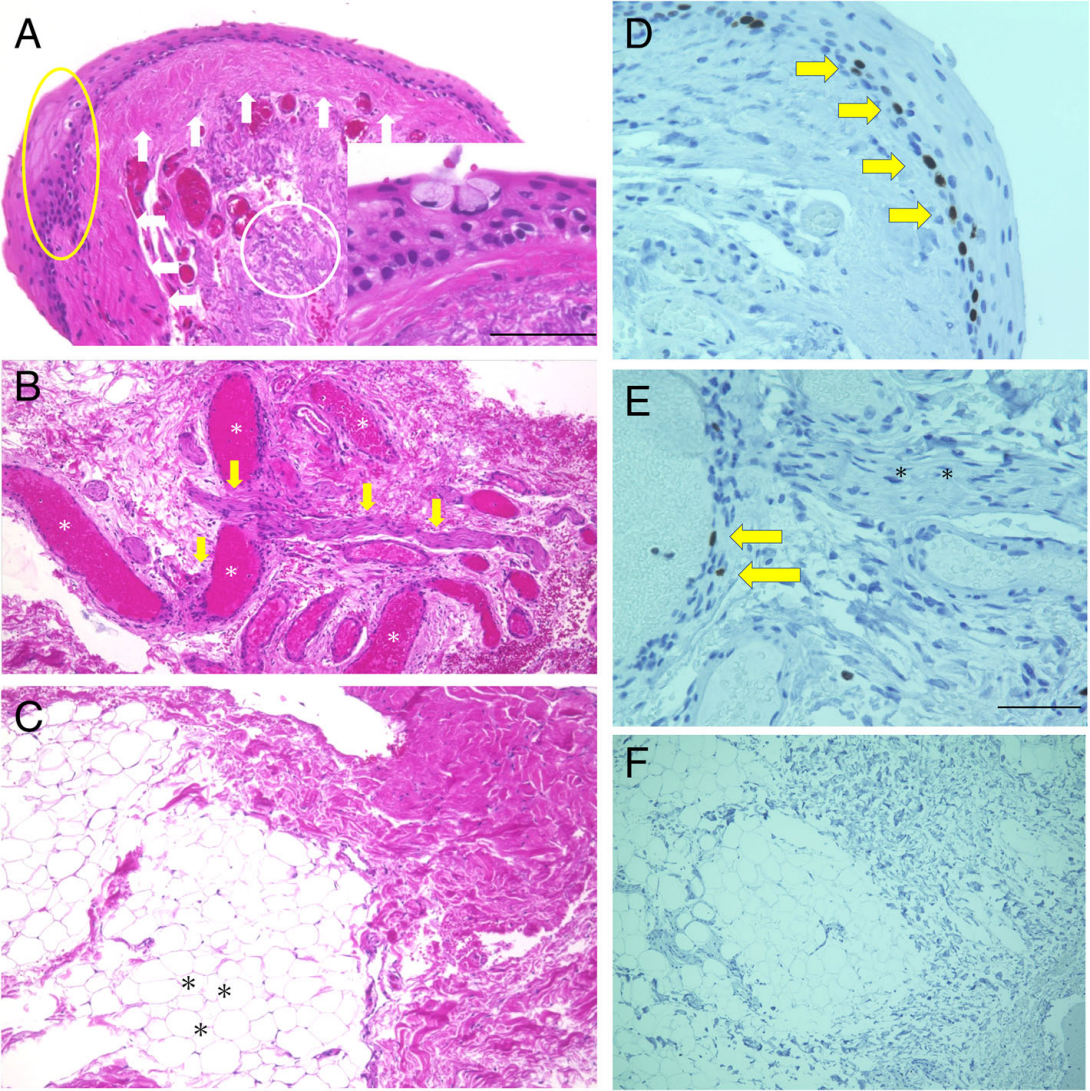
Histopathological findings (**a**-**c**) and immunoreactivity for Ki67 (**d**-**f**) in the excised pterygium-like tissue. **a** The head of the excised tissue was covered by stratified squamous metaplasia (yellow circle). Dense collagenous tissue was located beneath the epithelium (white arrows), where a collection of degenerated elastic fibers (white circle) was intermingled. Some goblet cells were contained in the epithelium (insert). A bar indicated 50 μm. Hematoxylin and eosin (H&E) stain. **b** The body of the excised tissue was rich in dilated blood vessels (white asterisks). A peripheral nerve was found in the subepithelial stroma (yellow arrows). H&E stain. **c** The body of the excised tissue also contained mature adipose cells (black asterisks) and collagen fibers in the subepithelial stroma. H&E stain. **d** A number of Ki67-immunopositive cells were located in the pterygial epithelium (yellow arrows). A bar indicated 50 μm. **e** Ki67 was immunoreactive in several endothelial cells in the neovessels (yellow arrows) but not in the peripheral nerve (asterisks). A bar indicated 50 μm. **f** Immunoreactivity for Ki67 was not detected in the adipose and collagen tissues

The head of the excised tissue was histologically covered with stratified columnar epithelium mixed with goblet cells and squamous metaplasia (Fig. 2a, yellow circle, and inserted figure). Dense collagenous tissue was located beneath the epithelium (Fig. 2a, white arrows), where a collection of degenerated elastic fibers (Fig. 2a, white circle) was intermingled. Unexpectedly, the body of the excised tissue contained a peripheral nerve (Fig. 2b, yellow arrows) in the subepithelial stroma surrounded with a number of dilated neovessels (Fig. 2b, white asterisks) and collagen fibers. The body of the excised tissue also contained mature adipose cells (Fig. 2c, asterisks) and collagen fibers. Immunohistochemistry for Ki67, a cell proliferation marker, was further confirmed. Briefly, the slide was dewaxed, rehydrated, and rinsed in phosphate-buffered saline twice for 10 min. As a pretreatment, microwave-based antigen retrieval was performed in 10 mM citrate buffer (pH 6.0). The slide was treated with 3% hydrogen peroxide and normal goat serum. Sections were incubated with anti-Ki67 antibody (Mib-1, DAKO). Positive signals were visualized using 3, 3′-diaminobendizine as a substrate. Hematoxylin staining was conducted for the nuclear staining. Cells were examined using a Biorevo BZ-9000 microscope (Keyence, Osaka, Japan). Ki67 was immunopositive in the nuclei of pterygial epithelial cells and neovascular endothelial cells (Fig. 2d-e, arrows) but not in the connective tissue, the adipose tissue (Fig. 2f) or the peripheral nerve (Fig. 2e, asterisks).

## Discussion and conclusions

In the present case of pterygium-like proliferation, its temporal location and marked thickness with abundant adipose droplets were atypical of primary pterygium. However, the pathological findings of this case included the presence of goblet cells in the epithelial region with squamous metaplasia, subepithelial neovascularization, and elastic degeneration, all of which were typically found in primary pterygium. In contrast, the other pathological findings such as prominent subepithelial and stromal accumulation of collagen fibers, adipose tissue formation, and presence of a peripheral nerve were considered characteristic as limbal dermoid but not pterygium.

Although the pathological finding of degenerative elastic fibers, typically found in primary pterygia, was theorized to reflect chronic ultraviolet exposure, the clinical findings were atypical possibly due to modification with postoperative dermoid remnants. Immunohistochemistry for Ki67 further confirmed the presence of proliferating cells in the pterygial epithelium and neovessels, but not in dermoid components. In consistence with the widely recognized non-proliferating nature of congenital limbal dermoid, the currently observed postoperative remnants showed no proliferative tendency even under the biological environment in which pterygium developed with massive cell proliferation.

In conclusion, the development of the present case would thus be attributable to the etiology common to primary pterygium but modified to some extent by non-proliferative residual dermoid tissue.

## Data Availability

Not applicable.

## Abbreviations

BCVA: Best-corrected visual acuity
OS: Oculus sinister
